# Preventive Effect of Novel Bacterial Polysaccharide and Animal Splenic Protein as Natural Adjuvants on Animal Model of Asthma

**Published:** 2012

**Authors:** Majid Mirsadraee, Saleh Mohaghegh Hazrati, Mohammad Reza Khakzad, Kamran Ghafarzadegan, Mohhamad Hosein Boskabady

**Affiliations:** 1 *Department of Internal Medicine, Medical School of Islamic Azad University- Mashhad Branch, Mashhad, Iran*; 2 *Mohaghagh Hazreati Immunology and Biotechnology Researches Centre, Tehran Iran*; 3 *Department of Immunology, Medical School of Islamic Azad University, Mashhad Branch, Mashhad, Iran*; 4 *Department of Pathology Razavi Hospital, Mashhad, Iran*; 5 *Mashhad University of Medical Science, Mashhad, Iran*

**Keywords:** Asthma, Interleukin-4, Immunomodulation Interferon-gamma, Prevention and control, Adjuvants, Immunologic

## Abstract

**Objective(s):**

Two new adjuvants from natural animal lipids (G2) and bacterial polysaccharide extracts (PC) were previously prepared by our group and showed a reduction in tracheal responsiveness. The aim of this study was to evaluate the preventive effect of recently introduced natural products (G2 and PC) on the development of asthma.

**Materials and Methods:**

Asthma was induced using a standard method in four groups of BALB/c mice. A non-sensitized control group was also included in order to be compared with treated groups. Three groups were premedicated with novel agents named G2, PC, and a combination of these two for 20 days before starting the induction of asthma. Bronchoalveolar lavage fluid (BALF) was collected and analyzed for inflammatory cells. Interferon-γ, and IL-4 and the histopathological of both lungs were also evaluated.

**Results:**

In all pretreated groups, the inflammatory cells infiltration especially eosinophils and smooth muscle hyperplasia decreased significantly. BALF cytology also showed significant decrease in eosinophil count in all pretreated groups. There was a significant increase in the BALF and serum INF-γ in all pretreated groups but the combination of G2/PC was more effective. BALF IL-4 decreased significantly in the group pretreated with a combination of G2 and G2/PC (4.11±0.86 and 4.02±0.52 pg/ml in G2 and G2/PC, respectively). Serum IL-4 in the PC group was significantly higher than the sensitized control**.**

**Conclusion:**

G2 and PC may effectively prevent asthma development by activation of the type 1 T helper system.

## Introduction

Previous studies have shown that the activation of the T helper 2 system and its mediators can affect asthma in many ways such as airway inflammation, mucus secretion, and airway hyper-responsiveness (1). Considering the hygiene theory, many investigators have attempted to prevent asthma by inducing the activity of the T helper 1 system by natural immunomodulators (2). In addition, probiotics were known as cultures of potentially beneficial bacteria that affect the host by enhancing the microbial balance and therefore restore the normal intestinal permeability, gut microecology, and reducing the generation of pathologic proinflammatory cytokines (3).

Two different adjuvants from natural animal lipids (G2) and bacterial protein extracts (PC) were previously prepared by our group, which had been used on a murine model with adenocarcinoma type of ductal breast cancer (4). The results showed that these adjuvants could control and destroy tumors up to 42% and significantly increase the survival of cancer-induced mice (4). According to the above-mentioned study and phase I studies on cancer patients, G2 and PC adjuvants have been registered in the Iranian Patent Office as immune system activator vaccines. Thereafter, these agents were used to modulate immune responses (5). The most recent study on these drugs showed reduction in tracheal responsiveness with G2 and G2+PC (6). In this regard, further research is required to evaluate the potential effect of these agents on asthma.

The objective of this study was to evaluate the effect of G2 and PC on the prevention of asthma and its proposed mechanism in an animal model of asthma.

## Materials and Methods

***Sensitization of animals***

This experimental study was conducted on BALB-c mice and the experiment was performed once in the specific pathogen free facility of Zakaria Research Centre, Medical School of Islamic Azad University, Mashhad Branch. Thirty male BALB/c mice, approximately 6-8 weeks (35-40 g) were entered into this prospective study. Animals were divided into five groups of six mice. Animals were sensitized with ovalbumin in four groups (OA, Sigma grade 5). One group was not sensitized and served as the non-sensitized control group. Sensitization of animals was performed in 6-week old mice by intraperitoneal injection of OA (10 mg as antigen) and aluminum hydroxide (2 mg, as an antibody producing adjuvant) for once a week up to three weeks. After three weeks, an aerosol of OA 0.1% was administered in a closed chamber (dimensions 40×40×70 cm) using a compressor nebulizer (Omron CX3, Japan, particle size 3-5 µm and output of 5 l/min) for 20 min a day for eight times up to day 75. After this period, the mice were sensitized and coughing was a symptom that showed successful sensitization of animals. In three groups of animals, novel adjuvants including G2, PC, and their combination were injected (0.4 ml subcutaneous injection) daily for 20 days before the beginning of the sensitization.

***Protocols***

At the end of the procedure, the animals were anesthetized with 44 mg/kg intraperitoneal ketamin and a blood sample was taken. A tracheostomy was then performed from a cricothyroid membrane and bronchoalveolar lavage fluid (BALF) was collected using a 0.4 sterile PBS and a 0.9 mm catheter. Four BALF samples were taken for the evaluation of inflammatory cells (total and differential WBC) and interleukines. BALF was centrifuged and the remnant was spread on the slides and stained by May-Grunwald-Gimsa stain. Total and differential inflammatory cell count was calculated among 300 cells. Interleukin-4 (IL-4) and interferon-γ (INF-γ) in BALF and serum were measured using U-CYTech, (cytokine ELISA kit). Finally, autopsies were performed and both lungs were extracted and inflated with 0.5 ml formalin and a histopathology examination was performed. In this exam, certain criteria such as basement membrane thickening, smooth muscle hyperplasia, and eosinophilic infiltration were used to confirm asthma induction.

***Adjuvants***

G2 Adjuvant was prepared from Buffalo spleen lipid suspended in alcohol with a concentration of 20 µg/ml and registered as a patent in the Iranian Patent Office as: immune system activator vaccine (Innovation Register No: 36679, 28th of October, 2006). PC adjuvant is also a bacterial extract polysaccharide that has been derived from the culture of *Mycobacterium tuberculosis* H37Rv and registered as a patent in the Iranian patent office as: immune system activator vaccine (Innovation Register No: 36681, 28th of October, 2006). Their method of preparation was explained in our previous study (6). PC is water-soluble and in this study, it was used with a concentration of 2.5 µg/ml. These agents were known to be safe for human beings according to phase I of a clinical study in cancer patients (based on a private communication with Saleh Mohaghegh Hazrati).

***Ethical consideration***

The project was approved by the Ethical Committee of the Islamic Azad University of Mashhad and this study was reviewed by internal board of research department of Islamic Azad University - Mashhad branch. The usage of drugs in this trial was approved by the Ethical Committee of the Tehran University of Medical Sciences. Working with the animals during the study was performed according to animal rights laws and with minimal stress.

***Statistical analysis***

Normal distribution of the data was checked using the Kolmogrov-Smirnof test. Non-parametric analysis was used when normal distribution was not observed. Statistical analysis was done by the unpaired t test (two tailed) for evaluating differences of total inflammatory cells, IL-4, and INF-γ between sensitized and non-sensitized groups and between treated and sensitized groups. The Mann-Whitney U test was used to determine differences of inflammatory infiltration in the histopathology and the chi-square or Fisher's exact test was used for the evaluating difference between eosinophilic infiltration and submucosal muscular hypertrophy. Percentage of inflammatory cells, IL-4, and INF-γ in BALF and serum were compared between the treated groups by the ANOVA test. Epi Info 2007 (v3.4) and SPSS 14 software were used for statistical analysis. Significance was accepted at *P*< 0.05.

## Results

**Figure 1:**
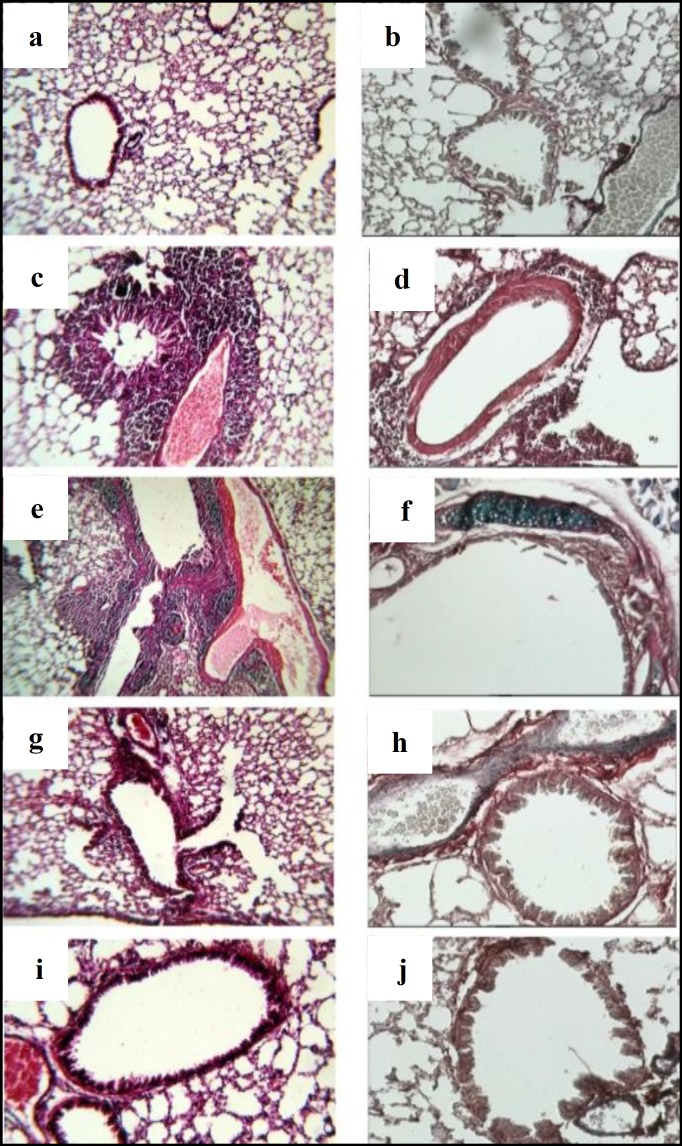
1a and 1b show a normal lung with H&E stain of normal non-sensitized animals, 1c: Histological changes of pulmonary tissue in ovalbumin-induced sensitized non-treated BALB/C, characterized by intensive inflammation caused by recruitment of eosinophils, neutrophils, macrophages, and lymphocytes along with mucous edema and smooth muscle hypertrophy (H&E), 1d: Masson's trichrome stain shows the remodelling and increased smooth muscle layer of the airway, 1e: High power details of the lung after treatment with G-2. It shows marked inflammatory cell infiltration including eosinophils within the airways and blood vessels (H&E), 1f: The Trichrome stain in the G2 group shows marked smooth muscle hyperplasia, 1g: Lung histology after treatment with PC reveals marked reduction in inflammation and edema (H&E), 1h: The Trichrome stain in this group demonstrated inconspicuous smooth muscle layer hyperplasia, 1i: Lung parenchyma after treatment with G2 + PC. The pathologic changes of asthma are almost completely disappeared and returned to normal (H&E), and 1j: Normal smooth muscles are also noted in the bronchi (Trichrome stain)

One mouse died for unknown reasons in the group that received combined G2 and PC (autopsy was unremarkable). The histopathologic study of the sensitized control group showed increased mucosal inflammatory cells (predominantly eosinophilic) (Mann-Whitney U=0.001, *P*=0.002 and Fisher exact=0.007, respectively) (Figure 1c) and smooth muscle hyperplasia (Figure 1d) that was significantly more than the non-sensitized control group (Figures 1a and 1b) (Fisher exact=0.03(. Collagen deposition was not observed in any of the groups (Figures 1b and 1d).

The PC and G2+PC pretreated groups showed low submucosal cellular infiltration that were not significantly different from the non-sensitized group (Figures 1g and 1i), but in the G2 group, the cellular infiltrate was significantly higher (Figure 1e) (Mann-Whitney U= 6, *P*= 0.02). Comparison of the cellular infiltrate between the sensitized group and G2 and G2+PC groups also showed no significant difference but the PC group showed significantly lower cellular infiltrate (Mann-Whitney U=1.5, *P*=0.005). Eosinophilic infiltration was reduced in all pretreated groups but the difference was not statistically significant except in G2+PC (Figure 1i) (Fisher=0.01). Smooth muscle hyperplasia in G2 was not significantly different from the sensitized group (Figure 1f) but this finding decreased significantly in the PC and G2+PC groups (Figure 1h and 1j) (Fisher exact= 0.01).

***Inflammatory cells in BALF***

Total inflammatory cell count in BALF was higher in the sensitized control group (1895±312 /ml) compared with the non-sensitized control group (145±73 per ml,* P*< 0.001) (Table 1). 

In PC and G2 pretreated groups, the mean of inflammatory cells were 430±155 and 1310±360 /ml, respectively, which were significantly lower than the sensitized control group (t=10.24, *P*= 0.001 and t= 3.1, *P*= 0.014, respectively), but still significantly higher than the non-sensitized group (t=7.7, *P*= 0.001 and t= 4.1, *P*= 0.002, respectively). In the PC+G2 group, the difference in the total inflammatory cell count with the sensitized control group was not significant. Total inflammatory cell count in the PC group was also significantly lower than the G2 and combined groups (t= 5.4, *P*= 0.001 and t= 4, *P*= 0.003, respectively).

***Differential counts of inflammatory cells***

Differential counts of inflammatory cells of four major groups are shown in Table 1. 

**Figure 2:**
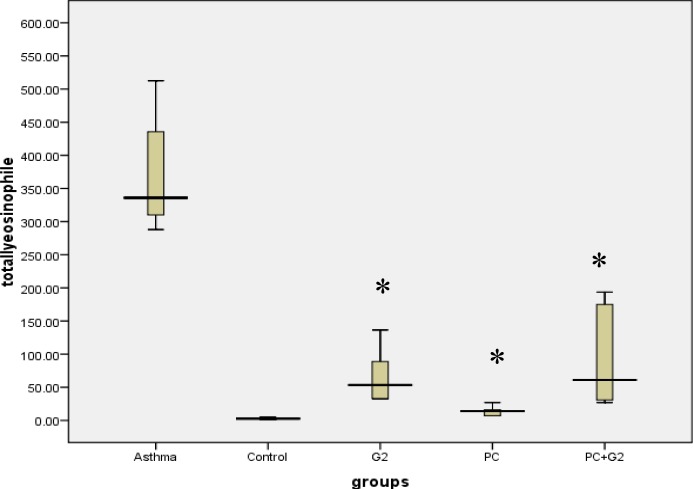
Comparison of total eosinophile count in non-sensitized, control sensitized, and treatment groups consisted of PC, G2, and the combination of both

In comparison with the non-sensitized control group, the neutrophil absolute count increased in all groups, especially in the G2 and G2+PC group which was significantly higher than the PC group (Table 1). Eosinophile in the sensitized control group was significantly higher than the non-sensitized control group and all treated groups. Comparison of treated groups showed that eosinophile was not significantly different between groups, but it decreased significantly in all treatment groups (F=1.2, *P*= 0.9) (Figure 2). 

**Table 1:** Total WBC, absolute and percentage of inflammatory cells in bronchoalveolar lavage of sensitized mice pretreated with G2 and PC and their combination (PC+G2), (n= 6 for each group)

	Total cells (per ml)	Neutrophil (% of total Total/ml)	Mononuclear (% of total Total/ml)	Eosinophil (% of total Total/ml)
Non-sensitized	143±73	40.04±2.16* 60±29.6	57±1.8 83±42	1.8±0.15* 2.7±1.7*
Sensitized control	1895±312†	26.5±2.81† 499±76.4	54±2 1026±197†	19.5±3.08† 369±86.3†
PC	430±155†*	23.6±4.2† 102±45.6	73±4.8*† 313±111*	3.5±1.7* 14.2±7.2*
G2	1310±360†*	45.8±11.9* 586±179†	48±15 657±332†	5.3±3.2* 66.3±40.3*
PC+G2	2008±956†	54.2±10.7†* 1129±644*†	39±4.8†* 738±311†	4.4±2.5* 97±80.5*

Data is shown as mean ± SD. *: significant difference of treated groups with sensitized control group, †: significant difference of treated groups with non-sensitized control group, BALF: Bronchoalveolar lavage fluid, * and † *P*< 0.05

**Figure 3:**
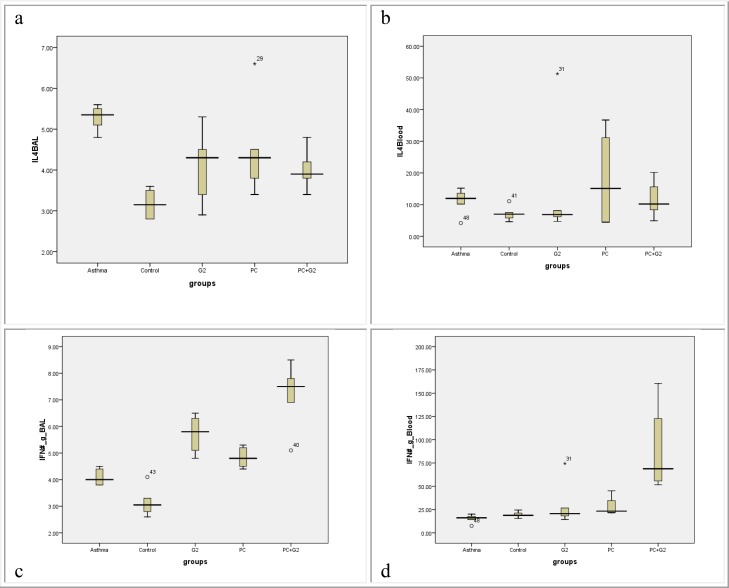
Comparisons of BALF IL-4 (a), serum IL-4 (b), BALF INF-γ (c), and serum INF-γ (d) in non-sensitized, control sensitized, treated with adjuvant PC, G2, and both adjuvant (PC+G2) BALB-c mice, (six mice for each group, data are shown as mean±SD and pg/ml unit of measurement)

**Table 2:** Mean and standard deviation of interferon gamma (IFN-γ) and IL-4 in bronchoalveolar lavage fluid and serum of sensitized mice pretreated with G2 and PC and their combination (PC+G2), (n= 6 for each group)

	IL-4 BALF (pg/ml)	IFN-γ BALF (pg/ml)	IL-4 Serum (pg/ml)	IFN-γSerum (pg/ml)
Non-sensitized	3.16±0.33	3.15±0.53	7.16±2.19	19.7±3.3
Sensitized control	5.28±0.3	4.08±0.3	11.18±3.82	15.3±4.3
PC	4.48±1.11	4.84±0.4*	17.82±15.9*	28.2±11.2*
G2	4.11±0.86*	5.71±0.68*	14±18.3	29.1±22.5*
PC+G2	4.02±0.52*	7.16±1.2*	11.8±6.05	91.8±47.7*

Data are shown as mean±SD. All mediators showed significant difference between treated groups and non-sensitized control group (* showed comparison of treated groups with sensitized control group), IL-4= Iinterleukin-4, IFN-γ= Interferon-γ, *= *P*< 0.05

Mononuclear absolute count increased significantly in the sensitized control group and all treated groups, but in the treated groups it was lower than the sensitized control group (the differences was only significant for PC). In summary, in the sensitized control group, total inflammatory cells increased, and treatment with adjuvants resulted in decreased total count and all subgroup cells (most prominently in eosinophils), except for the neutrophils in the combination therapy group. Analysis of single and combination therapy groups showed that cellularity in the PC group was more similar to the control non-sensitized group than the other treated groups. 

***IL-4***

BALF and serum IL-4 increased significantly in all groups, in comparison with the non-sensitized group (Figure 3, Table 2). G2 and the combination of G2 and PC were able to decrease the BALF IL-4 significantly (compared with the sensitized control) (t= 3.18, *P*= 0.018 and t= 4.77, *P*= 0.003, respectively). However, with a marginal result, IL-4 in the PC group was not significantly different from the sensitized control group (Figure 3, Table 2). Differences of IL-4 between pretreated groups were not significant.

Serum IL-4 in the PC group was significantly higher than the sensitized control group (F statics= 23, *P*= 0.001) (Figure 3, Table 2), but the differences between the other treatment groups and sensitized control group were not significant.

***Interferon γ***

BALF INF-γ in the sensitized control group and all treated groups showed significant increase in comparison with the non-sensitized group (Figure 3, Table 2) (*t* test ranged from 3.72 up to 7.2, *P*< 0.006). All immunomodulators in the three pretreated groups were able to increase the BALF INF-γ, compared with the sensitized control group (*t* test ranged from 3.4 up to 5.3, *P*< 0.01). Comparison between the pretreated groups showed that BALF INF-γ in G2 and the combination of G2 and PC were significantly higher than the PC group (t= 2.62, *P*= 0.03 and t=3.8, *P*=0.013, respectively) (Figure 3, Table 2). 

Serum INF-γ showed a significant increase in animals pretreated with G2 and PC compared with the sensitized and non-sensitized control groups (Figure 3, Table 2). The combination of G2 and PC was able to increase serum INF-γ significantly more than all other groups (t test ranged from 2.5 up to 3.8, *P*< 0.039) (Figure 3, Table 2).

[desired position for table 2]

## Discussion

The incidence of asthma has been increasing over the past decades (1) . Previous studies introduced primary and secondary measures to prevent asthma (8). Measures such as avoidance of exposure to environmental pollution, tobacco smoke exposure, and exclusive breastfeeding was recommended for prevention of asthma induction (9). In addition, early life dietary intervention with vitamins and polyunsaturated fatty acids has conflicting results in this regards (10). Because the T helper 2 system is thought to play a key role in asthma, many studies were conducted to evaluate preventive drugs that block T helper 2 or its mediators (11). According to the hygiene theory, immunomodulatory methods were investigated by the administration of microbial adjuvants and probiotics (12), especially lactobacillus species to divert T helper 2 response to T helper 1 response (13). Another useful immunomodulatory therapy was specific immunotherapy with allergen extracts (14) and the humanized monoclonal anti-IgE antibody (15). Recently, macrolide antibiotics were used as immunomodulatory drugs resulting in an improvement in clinical outcome (16). Immunomodulatory treatments were reported to be more promising than previous controller drugs. They can be curative due to inhibition of underlying immune pathology (12). 

In this study, the sensitization (asthma induction) in mice was prevented by premedication with two new natural immunomodulatory drugs. In the sensitized control group, total inflammatory cells increased in BALF and the analysis of subgroups showed that neutrophils, mononuclear cells, and eosinophil absolute count increased. In this group, BALF IL-4, and INF-γ increased, but alteration of IL-4 and INF-γ in serum was not significant.

In groups that were premedicated with G2 and PC, BALF total inflammatory cells, especially eosinophils as the hall mark of asthma, decreased. The combination of G2 and PC did not affect the total inflammatory cells in BALF. In this group, neutrophil increment was a reflection of inflammation in sensitized mice as described before (17), but reduction of eosinophil percentage was detected in this group. T helper 17 and IL-17 might be the mechanism to increase neutrphils. Future studies on IL-17 can reveal more information about mechanism of these agents.

In premedicated sensitized mice, G2 and the combination of G2 and PC were able to decrease the BALF IL-4 and all three treated groups were able to significantly increase the BALF INF-γ compared with the sensitized control group. It means that G2 and to a lesser extent PC may prevent asthma by suppressing the T helper 2 system via increasing the activity of the T helper 1 system.

 In addition, some variability between the groups was seen. The combination of G2 and PC was the only agent that could increase the INF-γ in serum and G2 could increase BALF INF-γ more effectively than PC.

Another study by the Euromieh Training and Research Health Centre (in western Iran) showed that in the BALB/c mice models of asthma, percentage of eosinophils and IgE in BALF and serum decreased with  pretreatment of mice with G2 and PC (unpublished data). Furthermore, the preliminary result of a study in Tabriz (Iran) has shown that these immunomodulatory drugs could lead to the treatment of children and adult asthma patients without any obvious side effects. Although previous reports suggested the ability of oral probiotics for preventing asthma (18), the current study showed more promising results toward the prevention of asthma especially by increasing INF-γ.

Concerning eosinophilic inflammation in asthma, the results of the present study clearly showed reduction of eosinophils in BALF of sensitized animals treated with the adjuvants as well as a reduction in pathological changes of the lung. Therefore, the results indicated a therapeutic effect of adjuvants on eosinophilic inflammation in asthma. 

## Conclusions

G2 and PC may be used as supplementary drugs for prevention of asthma in families with a history of asthma.
